# Dr. Rush P. Brown

**Published:** 1914-05

**Authors:** 


**Dr. Rush P. Brown,** N. Y. University 1873, was found dead in bed April 9, at his home in Addison. He was born in Springfield, Pa., 1847. After his graduation he spent his life in Addison.

